# A 3–4 h oocyte retrieval-ICSI interval optimizes clinical outcomes for women over 40 years

**DOI:** 10.1038/s41598-023-41397-7

**Published:** 2023-09-12

**Authors:** Jingbo Chen, Yanfang Wang, Cong Fang, Tingting Li

**Affiliations:** 1 Reproductive Medicine Research Center, Sixth Affiliated Hospital of Sun Yat-Sen University, NO.17, Shougouling Rd, Guangzhou, 510275 China; 2 Department of Reproductive Medicine, Guangdong Provincial People’s Hospital (Guangdong Academy of Medical Sciences), Southern Medical University, Guangzhou, 510080 China; 3 Guangdong Engineering Technology Research Center of Fertility Preservation, NO.17, Shougouling Rd, Guangzhou, 510275 China; 4 Biomedical Innovation Center, The Sixth Affiliated Hospital, Sun Yat-sen University, Guangzhou, China

**Keywords:** Biological techniques, Medical research

## Abstract

To explore an appropriate time interval between oocyte retrieval and intracytoplasmic sperm injection (ICSI) for optimal embryological and clinical outcomes in ICSI cycles over 40 years of maternal age. A retrospective analysis of 1476 ICSI fresh cycles from women aged over 40 years, was performed at the Reproduction Medicine Research Center of the Sixth Affiliated Hospital of Sun Yat-sen University, between December 2013 and August 2020. The fertilization rate and clinical pregnancy rate were the main outcomes. Multivariate linear regression and logistic regression analysis of factors showed that fertilization rate (*P* = 0.024) and clinical pregnancy rate (*P* = 0.011) were significantly associated with oocyte pick up (OPU)-ICSI interval. A longer OPU-ICSI interval (no more than 6 h) was associated with a higher fertilization rate but significantly decreased the clinical pregnancy rate when the OPU-ICSI interval was over 4 h (*P* < 0.05). The optimal OPU-ICSI interval is between 3 and 4 h for excellent embryological and clinical outcomes in ICSI cycles over 40 years of maternal age.

## Introduction

In 1992, intracytoplasmic sperm injection (ICSI) was born to cope with severe male factor infertility in whom IVF had failed^1^. Optimal timings of ICSI procedures, including HCG trigger, oocyte pick up (OPU), denudation (DN), and ICSI, are closely related to successful ICSI outcomes^2,3^. Although the ICSI techniques have been completely standardized, the precise timings of ICSI procedures have no common standard.

In natural human menstrual cycles, the onset of luteinizing hormone (LH) surge, deemed as an indicator of ovulation, usually occurs 24–56 h earlier^4^, and the most common LH surge -ovulation interval is 34–38 h^5,6^.

Several studies have found that the time interval of oocytes being fertilized is much longer than that of their capacity to develop into viable embryos: oocytes inseminated over 56 h after hCG administration fertilize only at a slightly lower rate than those inseminated approximately 40 h after hCG administration; however, the implantation rate of these embryos declines to nearly zero over the same period^7,8^. What’s more, it is well known to all that ovarian reserve and retrieved oocytes will reduce as the maternal age increases. Oocytes and embryos are much more precious for the advanced maternal age (AMA) population. Therefore, the timing of performing ICSI is one of the vital factors determining embryo viability, especially for advanced-aged women.

A few studies focusing on the effect of ICSI procedure timings on clinical outcomes were published^9,10^, with discrepancies in the conclusions. Some studies reported that a preincubation period for 2–6 h between oocyte retrieval and in vitro fertilization (IVF) or ICSI improved MII oocyte rates^11,12^, fertilization and pregnancy rates^9,12–15^, and embryo qualities^9–16^. Although a too-long oocyte preincubation (9–11 h) before ICSI is supposed to have adverse effects on embryo qualities^16^, probably due to oocyte aging, other studies showed different results that preincubated oocytes had no statistical significance in the fertilization^17,18^ or the pregnancy rates^12,16^ during ICSI cycles. All the studies mentioned above were based on young patients with an average age of less than 38 years old. No exact optimal ICSI timing has been concluded till now, especially in the advanced maternal age populations over 40 years old.

The objective of this study was to retrospectively analyze microinjection timings on ICSI outcomes in a specific population aged over 40 years, to find out the optimal time for ICSI to improve clinical outcomes in this population.

## Methods and materials

### Study population and ethical approval

We retrospectively analyzed 1476 ICSI cycles from women, aged over 40 years old, at the Reproduction Medicine Center in the Sixth Affiliated Hospital of Sun Yat-sen University, between December 2013 and August 2020. Considering that the study covered a long time, there were some patients, who underwent more than one ICSI cycles. Although some ICSI cycles were from the same patient, the parameters of the lCSI cycles would not be identical. For example, age, AMH level, AFC, BMl, etc. of the same patient were changed with time. Therefore, the ICSI cycles were considered independent of each other. To avoid bias in the analysis, the exclusion criteria in this study were: (a) the patients who used frozen-thawed oocytes or donor oocytes for fertilization; (b) women or partners with genetic diseases requiring preimplantation genetic testing (PGT); (c) males with obvious sperm morphology abnormality or with sperms from either testicular or microsurgical epididymal sperm aspiration. All patients signed written informed consent on the anonymous use of their data. This study was approved by the Institutional Reviewer Board of Sixth Affiliated Hospital of Sun Yat-sen University (2017ZSLYEC-016S). All methods were carried out in accordance with relevant guidelines and regulations.

### Protocols for controlled ovarian stimulation (COS)

Our controlled ovarian stimulation (COS) protocols in this study are the routine protocols in our center, containing GnRH agonist long protocol, GnRH agonist short protocol, GnRH antagonist protocol, luteal phase ovarian stimulation, and progestin-primed ovarian stimulation (PPOS) protocol^13^. Ovarian stimulation was carried out using recombinant follicle-stimulating hormone (r-FSH) (Gonal-F, Serono, Sweden) combined with gonadotropin-releasing hormone (GnRH) agonist (Decapeptyl; Ferring, Kiel, Germany) and antagonist (Cetrotide; Merck Serono, Darmstadt, Germany). COS protocols and daily doses of FSH injection of the patients in our research were performed according to female ages, ovarian reserve, and various reactions to ovarian stimulation in previous cycles. Follicular developments were monitored with transvaginal ultrasound scanning. The Human Chorionic Gonadotropin (hCG) (Lizhu Pharmaceutical Trading Co., China) 4000–10,000 IU or triptorelin (Decapeptyl, Ferring pharmaceuticals, Germany) 0.1 mg and hCG (Lizhu Pharmaceutical Trading Co., China) 1000 IU were administered for ovulation triggering when one leading follicle reached a diameter of 18 mm or two leading follicles reached 17 mm. Normally, all accessible follicles above 10 mm were aspirated guided by transvaginal ultrasound when picking up oocytes. Then oocytes were cultured in a human tubal fluid (HTF) medium (Quinn’s Advantage fertilization HTF medium; Quinn’s, SAGE, USA), in 5–6% CO_2_ at 37 °C. The cumulus-corona-oocyte complexes obtained were incubated until the moment of ICSI.

### Timing control

Exact times including HCG trigger, OPU, and ICSI were recorded by the operators to establish a databank. We identified HCG trigger to OPU interval as T1, OPU to ICSI interval as T2, and HCG trigger to ICSI interval as T3. The detailed times, from HCG to ICSI, were analyzed to evaluate the influence of time intervals on ICSI outcomes.

### Outcome measure

In our research, we regarded the clinical pregnancy rate as the primary outcome and the fertilization rate as the secondary outcome. Fertilization rate was defined as the number of fertilized embryos obtained per oocyte injected multiplied by 100 for ICSI^19^. Transferrable D3 embryo was defined as no less than 4 blastomeres on day 3, at least slightly uneven size of blastomere and < 20% fragmentation. Implantation rate was defined as the number of gestational-sac(s) divided by the number of embryo(s) transferred. Biochemical pregnancy was defined as a positive serum β-HCG 15 days after transfer. Clinical pregnancy was defined as a visible embryo with a fetal heartbeat 5 weeks after transfer.

### Statistical analysis

Student-t test or Mann–Whitney U test was used for continuous variables. Results were expressed as mean ± SD. Chi-square was used for categoric variables. Correlation analysis, multivariate linear regression analysis, and logistic regression analysis were used to study the effect of intervals on embryological and clinical outcomes in ICSI cycles. All the data were analyzed with SPSS 26.0 (IBM, Armonk, NY, USA). *P* value less than 0.05 was defined as statistical significance. In addition, the graphs were prepared with GraphPad Prism version 5 (GraphPad Software, La Jolla, CA, USA).

### Ethics approval

This study was approved by the Institutional Reviewer Board of Sixth Affiliated Hospital of Sun Yat-sen University (2017ZSLYEC-016S). All methods were carried out in accordance with relevant guidelines and regulations.

### Consent to participate

Informed consent was obtained from all individual participants included in the study.

## Results

The study included a total of 1476 fresh ICSI cycles with maternal age over 40 years old between December 2013 and August 2020. Table 1 lists the basic demographics, cycle characteristics, laboratory and clinical outcomes of included cases. The mean age of women in the study was 42.44 ± 2.07 years (range 40–50 years). The mean BMI of women was 23.35 ± 2.85 kg/m^2^. The HCG-OPU intervals ranged from 635 to 3650 min (mean 2144.22 ± 120.73 min). The OPU-ICSI intervals ranged from 10 to 360 min (mean 176.29 ± 66.14 min).

**Table 1:** Basic demographics, cycle characteristics, laboratorial and clinical outcomes of ICSI cycles.

Parameter	Mean ± SD
Cycles	1476
Age (year)	42.44 ± 2.07
BMI (kg/m^2^)	23.35 ± 2.85
Basal FSH (IU/L)	9.24 ± 4.79
Basal LH (IU/L)	5.00 ± 2.85
Basal E2 (IU/L)	60.50 ± 109.14
AMH (ng/mL)	1.35 ± 1.40
AFC	6.22 ± 4.32
Total dose of Gn	1869.03 ± 953.35
Duration of Gn (days)	8.24 ± 2.93
No. of oocytes retrieved	4.84 ± 4.33
MII oocyte rate (%)	82.73 ± 20.39
Fertilization rate (%)	70.80 ± 34.06
Clinical pregnancy rate (%)	18.20
HCG-OPU interval (min)	2144.22 ± 120.73
OPU-ICSI interval (min)	176.29 ± 66.14
HCG-ICSI interval (min)	2320.51 ± 131.11

Data are presented as mean ± SD or n (%).

*BMI* body mass index, *AFC* antral follicle count, *Gn* gonadotropin, *OPU* oocyte pick-up, *ICSI* intracytoplasmic sperm injection.

Correlation analysis of T1–T3 associated with reproduction rates was shown in Table 2. T2 was significantly correlated with the fertilization rate (*P* = 0.002, rho = 0.374, effect size = moderate); T2 and T3 were significantly correlated with the clinical pregnancy rate (*P* = 0.046, rho = -0.597, effect size = large vs. *P* = 0.020, rho = -0.313, effect size = moderate). While there was no significant correlation of T1 with the fertilization rate or the clinical pregnancy rate.

**Table 2:** Correlation analysis between ICSI procedure timings and laboratorial and clinical outcomes in ICSI cycles.

Time interval	Fertilization rate		Transferable embryo rate		Good quality embryo rate		Biochemical pregnancy rate		Clinical pregnancy rate	
	rho	*P*	rho	*P*	rho	*P*	rho	*P*	rho	*P*
T1	− 0.039	0.106	− 0.053	0.042^b^	− 0.014	0.559	− 0.032	0.519	− 0.075	0.126
T2	0.374	0.002^b^	0.052	0.0498^b^	0.025	0.299	− 0.072	0.143	− 0.597	0.046^b^
T3	0.020	0.406	− 0.002	0.948	0.019	0.425	− 0.071	0.147	− 0.313	0.020^b^

rho: Spearman correlation coefficient.

T1: HCG-OPU interval.

T2: OPU-ICSI interval.

T3: HCG-ICSI interval.

^a^Correlation is significant < 0.001 level.

^b^Correlation is significant < 0.05 level.

The multivariate linear regression and logistic regression analysis related to fertilization and clinical pregnancy rates were shown in Table 3. The fertilization rate (*P* = 0.024, R^2^ = 0.160, effect size = moderate) and clinical pregnancy rate (*P* = 0.011, R^2^ = 0.360, effect size = large) were significantly related to T2 in ICSI cycles.

**Table 3:** Multivariate linear regression and logistic regression analysis between ICSI procedure timings and laboratorial and clinical outcomes in ICSI cycles.

Time interval	Fertilization rate			Transferable embryo rate		Clinical pregnancy rate		
	B	*P*	95% CI	B	*P*	B	*P*	95% CI
T1	–	–	–	− 0.015	0.579	–	–	–
T2	0.058	0.024^b^	0.058–0.058	0.006	0.834	0.996	0.011^b^	0.994–0.998
T3	–	–	–	–	–	–	–	–

T1: HCG-OPU interval; T2: OPU-ICSI interval; T3: HCG-ICSI interval.

^a^Correlation is significant < 0.001 level.

^b^Correlation is significant < 0.05 level.

**Figure 1:**
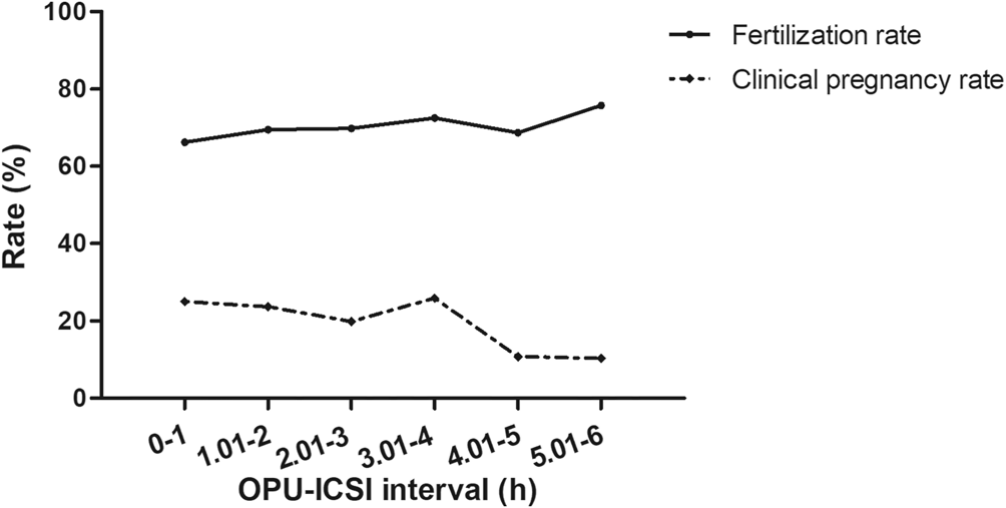
The overall association between the OPU-ICSI interval and fertilization rate and clinical pregnancy rate.

To get a detailed correlation, T2 was divided into nine groups: 0–1.00 h, 1.01–2.00 h, 2.01–3.00 h, 3.01–4.00 h, 4.01–5.00 h, 5.01–6.00 h. Table 4 lists the fertilization rates and clinical pregnancy rates at different T2 times for the nine groups. Within 6 h of T2, a prolonged T2 resulted in a raise of the fertilization rate—66.19% in group 1 (0–1 h), 69.48% in group 2 (1.01-2 h), and 75.73% in group 6 (5.01–6 h) (Fig. 1). While, the clinical pregnancy rate showed a similar trend to the fertilization rate within 4 h, but dropped to 10.81% when ICSI was performed 4 h after oocyte retrieval (Fig. 1).

**Table 4:** The overall association between the OPU-ICSI interval and fertilization rate and clinical pregnancy rate.

OPU-ICSI interval (h)	0–1.00	1.01–2.00	2.01–3.00	3.01–4.00	4.01–5.00	5.01–6.00
Fertilization rate (%)	66.19	69.48	69.79	72.53	68.66	75.73
Clinical pregnancy rate (%)	25.00	23.68	19.90	25.93	10.81	10.34

**Figure 2:**
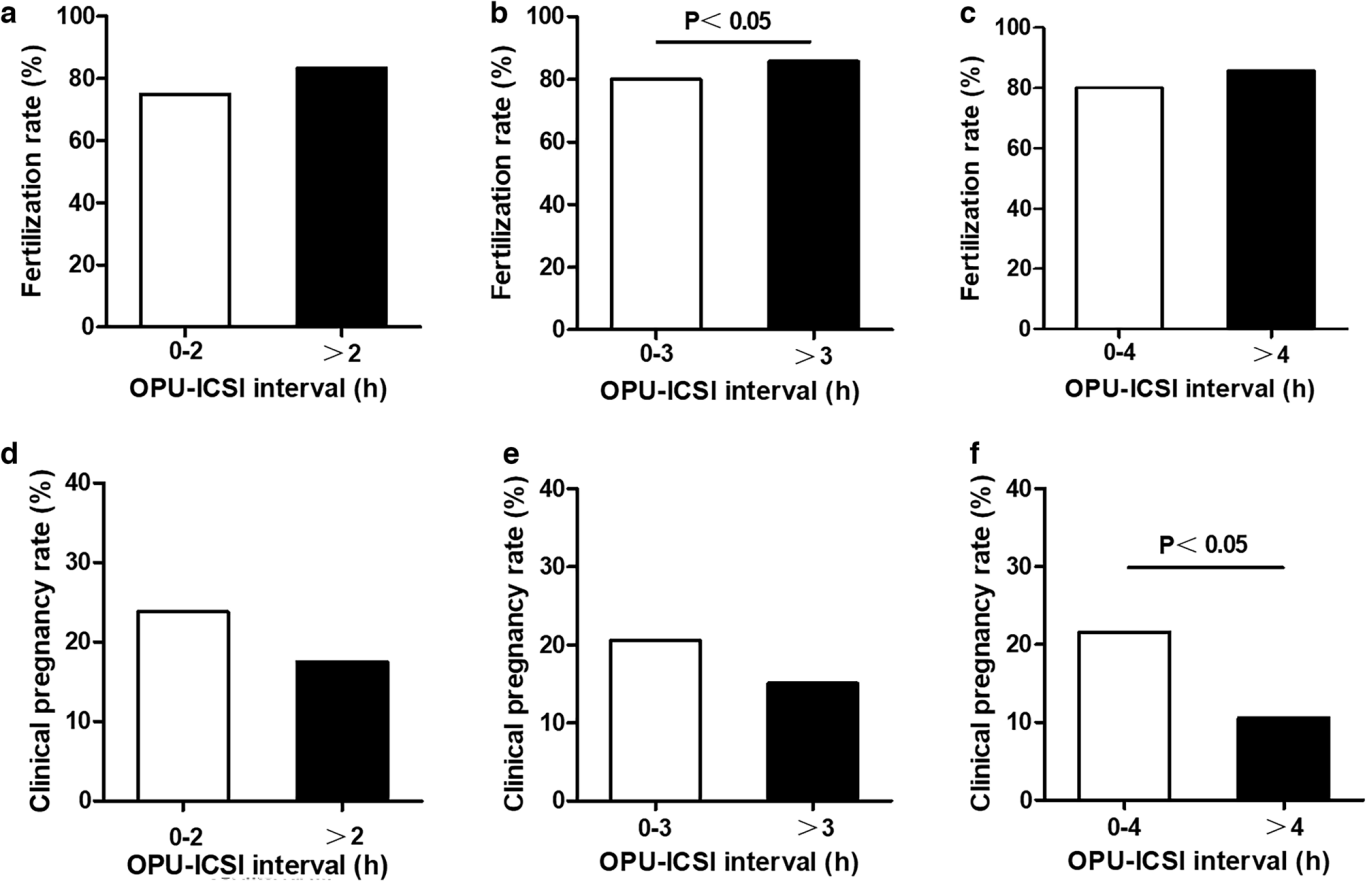
Fertilization rates with different OPU-ICSI interval cut-off points: (**a**) 2 h; (**b**) 3 h; (**c**) 4 h. Clinical pregnancy rates with different OPU-ICSI interval cut-off points: (**d**) 2 h; (**e**) 3 h; (**f**). 4 h. *P* value less than 0.05 was defined as statistical significance.

Hence, to find an appropriate cut-off point, 2 h, 3 h, and 4 h of T2 were chosen as cut-off points to determine the optimal ICSI time point. Figure 2 demonstrates that the fertilization rate was significantly higher in OPU-ICSI interval > 3 h compared to OPU-ICSI interval < 3 h; while the clinical pregnancy rate was significantly higher in OPU-ICSI interval < 4 h compared to OPU-ICSI interval > 4 h (*P* < 0.05). Thus, the optimal ICSI time point is 3–4 h after oocyte retrieval.

## Discussion

Our present study discovered that for AMA (≥ 40 years old) population, the OPU-ICSI interval can be an independent predictor for clinical outcomes in ICSI cycles. The fertilization rate increased continuously, reaching a peak of 75.73% when ICSI at 5–6 h post-OPU, but the clinical pregnancy rate was higher within 4 h post-OPU and then decreased sharply. The longer the OPU-ICSI interval, the lower the chances of pregnancy after fresh embryo transfer (ET). Therefore, according to our findings, it is recommended to perform ICSI within 4 h after OPU can optimize the clinical pregnancy rate in AMA (≥ 40 years old) population ICSI cycles.

However, limited studies are available on the relationship between the raised OPU-ICSI interval and the clinical outcomes. Most of them assumed that oocytes should be incubated in vitro surrounded by the corona and cumulus cells without analyzing the impact of both oocyte denudation and microinjection timings on ICSI outcomes^9–12,12,13,13–16^. Yanagida et al.^16^ recommended performing ICSI between 1 and 9 h after OPU (36–44 h post hCG) for that good-quality embryos were lower at 9–11 h than those within 9 h (P < 0.001) and pregnancy rate decreased significantly when at 9–11 h of preincubation time (7.7 vs 15.9%). Others found that fertilization rates increased continuously, reaching a peak of 84% > 6 h post-OPU (41 h post hCG), but the clinical pregnancy rate was higher within 2–6 h post-OPU (37–41 h post hCG) and then decreased^13^. Another study confirmed that the most appropriate incubation time before ICSI was 5–6 h (41–42 h post hCG) for that the fertilization rate increased slightly at 2–6 h and then decreased at 7–12 h of preincubation time, while the clinical pregnancy rate had a significant increase from 2 to 5 h after OPU and then dropping sharply^10^. A retrospective consecutive cohort study involving 1468 ICSI cycles showed increasing OPU-ICSI time increased the fertilization rate but decreased biochemical and clinical pregnancy after fresh ET^3^. These reports are indeed in agreement with our results that the fertilization rate increases but the clinical pregnancy rate decreases with time in culture.

A previous report demonstrated that it had no impact on fertilization and embryo quality no matter how long the time between OPU and denudation nor between denudation and ICSI is, but for fear that pronuclei may not be observed after an early injection, they advised delaying the injection whenever possible^17^. A large retrospective study including 3986 ICSI cycles also indicated no effect of OPU-ICSI time on either fertilization rate or pregnancy rates in young (≤ 35 years old) and fertile oocyte donors^20^. Later, a retrospective study concluded that 2–3 h preincubation with subsequent sperm injection may not increase MII rate but lead to optimal fertilization and implantation results also by separating the timing into two successive periods: OPU to denudation (T1) and denudation to microinjection (T2)^21^.

Kubiak et al. stated cytoplasmic and nuclear oocyte maturation were distinguishable procedures in the mouse. The process of cytoplasmic maturation was considered as the ability of the oocyte to be activated completely. If performing ICSI with immature oocyte cytoplasm, meiosis might be suspended and pronuclei might fail to form. In addition, Rienzi et al.^7^ reported that performing ICSI 3–12 h after OPU could obtain optimal fertilization rates when oocytes are undergoing full cytoplasmic maturation.

Since distinct genetic diseases greatly affect reproductive outcomes, we set exclusion criteria to avoid bias for the study. What’s more, another strength is that our research aims at infertile women over 40 years old, which provides an optimal timing point for performing ICSI exactly to aged women. In addition, detailed times from HCG to ICSI were recorded by the exact operators independently and we also adopt subdivisions of OPU-ICSI interval to proclaim the relationship between OPU-ICSI interval and fertilization rate and clinical pregnancy rate.

There were several limitations of the current study. For one limitation, we generally retrieved oocytes from patients with fewer follicles before we retrieved oocytes from patients with more follicles, which caused bias in associations between HCG-OPU intervals and the numbers of oocytes retrieved. For another, the implantation rate was not referred to as a primary outcome measure in our study due to the different number of embryos transferred in the enrolled patients. It is well known that the implantation rate was a more precise index for oocyte vitality, thus we will aim to explore a correlation between OPU-ICSI internal and embryo implantation potential in a well-designed single-embryo-transfer cohort study in our future work.

In conclusion, our results revealed that the increase in the fertilization rate might be due to the cytoplasmic maturation of immature oocytes and the stronger tendency of oocyte activation, which might also cause oocyte aging in vitro culture. OPU-ICSI interval can serve as an independent predictor for reproduction outcomes in ICSI cycles. The optimal time for ICSI is between 3 and 4 h after oocyte retrieval for ideal laboratory and clinical outcomes for women over 40 years.

## Data Availability

The datasets used and/or analyzed during the current study are available from the corresponding author on reasonable request.

## References

[1] Palermo G, Joris H, Devroey P, Van Steirteghem AC (1992). Pregnancies after intracytoplasmic injection of single spermatozoon into an oocyte. Lancet (London, England)..

[2] Mizuno S, Ishikawa Y, Matsumoto H (2019). The timing of cumulus cell removal for intracytoplasmic sperm injection influences the capability of embryonic development. Reprod. Med. Biol..

[3] Pujol A, García D, Obradors A, Rodríguez A, Vassena R (2018). Is there a relation between the time to ICSI and the reproductive outcomes?. Hum. Reprod..

[4] Temporal relationships between ovulation and defined changes in the concentration of plasma estradiol-17 beta, luteinizing hormone, follicle-stimulating hormone, and progesterone. I. Probit analysis. World Health Organization, Task Force on Methods for t. *Am. J. Obstet. Gynecol*. **138**(4), 383–390 (1980). 6775535

[5] Testart J, Frydman R (1982). Minimum time lapse between luteinizing hormone surge or human chorionic gonadotropin administration and follicular rupture. Fertil. Steril..

[6] Hoff JD, Quigley ME, Yen SS (1983). Hormonal dynamics at midcycle: A reevaluation. J. Clin. Endocrinol. Metab..

[7] Lundin K, Sjögren A, Hamberger L (1996). Reinsemination of one-day-old oocytes by use of intracytoplasmic sperm injection. Fertil. Steril..

[8] Morton PC, Yoder CS, Tucker MJ, Wright G, Brockman WD, Kort HI (1997). Reinsemination by intracytoplasmic sperm injection of 1-day-old oocytes after complete conventional fertilization failure. Fertil. Steril..

[9] Rienzi L, Ubaldi F, Anniballo R, Cerulo G, Greco E (1998). Preincubation of human oocytes may improve fertilization and embryo quality after intracytoplasmic sperm injection. Hum. Reprod..

[10] Falcone P, Gambera L, Pisoni M (2008). Correlation between oocyte preincubation time and pregnancy rate after intracytoplasmic sperm injection. Gynecol. Endocrinol. Off. J. Int. Soc. Gynecol. Endocrinol..

[11] Ho JY-P, Chen M-J, Yi Y-C, Guu H-F, Ho ES-C (2003). The effect of preincubation period of oocytes on nuclear maturity, fertilization rate, embryo quality, and pregnancy outcome in IVF and ICSI. J. Assist. Reprod. Genet..

[12] Isiklar A, Mercan R, Balaban B, Alatas C, Aksoy S, Urman B (2004). Impact of oocyte pre-incubation time on fertilization, embryo quality and pregnancy rate after intracytoplasmic sperm injection. Reprod. Biomed. Online..

[13] Dozortsev D, Nagy P, Abdelmassih S (2004). The optimal time for intracytoplasmic sperm injection in the human is from 37 to 41 hours after administration of human chorionic gonadotropin. Fertil. Steril..

[14] Trounson AO, Mohr LR, Wood C, Leeton JF (1982). Effect of delayed insemination on in-vitro fertilization, culture and transfer of human embryos. J. Reprod. Fertil..

[15] Khan I, Staessen C, Van den Abbeel E (1989). Time of insemination and its effect on in-vitro fertilization, cleavage and pregnancy rates in GnRH agonist/HMG-stimulated cycles. Hum. Reprod..

[16] Yanagida K, Yazawa H, Katayose H, Suzuki K, Hoshi K, Sato A (1998). Influence of oocyte preincubation time on fertilization after intracytoplasmic sperm injection. Hum. Reprod..

[17] Van de Velde H, De Vos A, Joris H, Nagy ZP, Van Steirteghem AC (1998). Effect of timing of oocyte denudation and micro-injection on survival, fertilization and embryo quality after intracytoplasmic sperm injection. Hum. Reprod..

[18] Jacobs M, Stolwijk AM, Wetzels AM (2001). The effect of insemination/injection time on the results of IVF and ICSI. Hum. Reprod..

[19] Johnson LNC, Sasson IE, Sammel MD, Dokras A (2013). Does intracytoplasmic sperm injection improve the fertilization rate and decrease the total fertilization failure rate in couples with well-defined unexplained infertility? A systematic review and meta-analysis. Fertil. Steril..

[20] Bárcena P, Rodríguez M, Obradors A, Vernaeve V, Vassena R (2016). Should we worry about the clock? Relationship between time to ICSI and reproductive outcomes in cycles with fresh and vitrified oocytes. Hum. Reprod..

[21] Patrat C, Kaffel A, Delaroche L (2012). Optimal timing for oocyte denudation and intracytoplasmic sperm injection. Obstet. Gynecol. Int..

